# SARS-CoV-2 Variants and COVID-19 in Bangladesh—Lessons Learned

**DOI:** 10.3390/v16071077

**Published:** 2024-07-04

**Authors:** Simon D. Lytton, Asish Kumar Ghosh

**Affiliations:** 1 SeraDiaLogistics, 81545 Munich, Germany; 2 Department of Virology, Dhaka Medical College Hospital, Dhaka 1000, Bangladesh; asish127kumar@gmail.com

**Keywords:** Bangladesh, COVID-19, SARS-CoV-2 variants, vaccination coverage, pandemic preparedness

## Abstract

The coronavirus infectious disease-2019 (COVID-19) in Bangladesh is a paradigm for how one of the most densely populated countries in the world, with 1270 people per square kilometer, managed to cope with the COVID-19 pandemic under extraordinary circumstances. This review highlights the SARS-CoV-2 variants in Bangladesh and the timeline of their detection in the context of the global experience with the management of vaccination and natural SARS-CoV-2 infection. The motivation to overcome the COVID-19 vaccine dilemma and track Bangladeshi SARS-CoV-2 sub-variants underscores the potential for a low-income country to excel in international medical science, despite having stressed health care services and limited availability of resources for SARS-CoV-2 testing and gene sequencing.

## 1. Introduction

The first cases of SARS-CoV-2 in Bangladesh were reported in March 2020 when the World Health Organization status of the COVID-19 global public health emergency was declared to be the COVID-19 pandemic. In May 2023, Bangladeshi government statistics reported over 2 million confirmed COVID-19 cases and 30,000 COVID-19-related deaths [1,2]. The emergence of SARS-CoV-2 variants with distinct phenotypes of transmissibility, COVID-19 severity and immune evasion was driven by the virus mutation rate and the recombination of genetically distinct viruses. While deleterious mutations are rapidly purged, the complex process of adaption, fitness and selection of advantageous variants is intimately tied to the immunity (vaccination and/or prior infection) and behavior within the human host population. The purpose of this review is to clarify the lessons learned regarding SARS-CoV-2 variants and COVID-19 in Bangladesh and present a perspective of the country’s unique medical and sociological responses to the pandemic. This review highlights Bangladesh’s coverage of vaccination and molecular surveillance of SARS-CoV-2 variants, which was more comprehensive than in many other countries.

## 2. The Will Power for Vaccination

The COVID-19 vaccination policies varied between countries according to priority groups, eligibility, availability and vaccine pricing, with some countries issuing mandatory vaccination for health care workers and other occupational categories [3]. High-income countries belonging to the Organization for Economic Cooperation and Development (OECD) largely completed their roll-out of vaccines by the end of 2021, whereas low-and middle-income countries completed their prioritization plans between May 2022 and December 2022 [4]. Irrespective of a country’s income, demographics or government policy, the vaccination of the general population faced two major dilemmas. First, public expectations of “full protection against COVID-19” based on the early waves of alpha and beta SARS-CoV-2 infection in the vaccinated health care setting were not reconcilable with the SARS-CoV-2 break-through infections by the delta and omicron infections that followed repeated immunizations in the general population [5,6]., Fully vaccinated individuals showed faster mean rate of viral decline with delta variant infections than did unvaccinated individuals [5], The community transmission of omicron infections in vaccinated individuals was associated with reinfections and persistent low viral loads in the nasal mucosa and upper airway [6]. The risk reduction measures of face masks, hand washing and social distancing in health care premises effectively controlled SARS-CoV-2 infections. Real-world data in Italy revealed that the vaccination of HCWs in COVID-19 units with high levels of viral circulation ensured that they were protected against hospitalized COVID-19. However, the daily COVID-19 cases among HCWs were two-fold to five-fold higher than the daily COVID-19 cases in the general population [3]. The second dilemma is the misconception that the viral RNA spike (S) protein sequence derived from the SARS-CoV-2 Wuhan-Hu-1 reference strain, the template for the development of commercial vaccines, was ineffective against new variants of concern (VOCs) [7,8]. Although mutations in the S-protein receptor-binding domain (RBD) led to evasion from neutralizing antibodies (nAbs) [9,10,11,12], the immune escape was primarily confined to the compartment of B-cell immunity. T-cell receptor (TCR) epitopes of the S protein were mostly conserved and marginally affected in SARS-CoV-2 variants [13,14]. Warnings that the virus evolution had rendered COVID-19 vaccines ineffective were largely incorrect. Vaccination proved effective at preventing symptomatic/severe COVID-19 [15,16] but not infections, which were mostly mild/asymptomatic during Omicron variant infections [17,18].

Despite the pervasive anti-vaccine sentiment in countries with resources for the development of vaccines [19,20,21], an estimated 65–80 percent of the European, American and Russian populations and more than 80 percent of the Canadian and Chinese populations had received two or more vaccine doses as of March 2023 [22,23].

Bangladesh began vaccination in February 2021 with the ChAdOx1-nCoV-19 vaccine known as Covishield, (Serum Institute India) facilitated by the Surokkha app, which required either a national identification or birth certificate number to receive and register for vaccination [24]. In July 2021, seven different COVID-19 vaccine preparations were in use amidst reported vaccine hesitancy of 32.5 percent in Bangladesh [25]. The Bangladeshi public received COVID-19 vaccines free of charge via donations of vaccine doses and funds from the WHO, the USA, Japan and the UK administered under the Expanded Program on Immunization (EPI) [26].

In August 2021, Incepta Vaccine Limited (IVL), the largest human vaccine manufacturing facility in Bangladesh, signed a memorandum of understanding with Sinopharm, China, for the production of the Sinopharm BBIBP-COVID-19 vaccine in Bangladesh. This collaboration and prudent vaccine diplomacy undoubtedly expedited the efforts of Bangladesh to produce COVID-19 vaccines and alleviated the country’s reliance on a single vaccine source [27]. In May 2022, Bangladesh ranked fifth in the Nikkei COVID-19 recovery index, an independent business assessment of the effective management of infection, vaccine roll-out and population mobility, making the country the leader in South Asia and surpassing the USA and several OECD countries [28,29]. The unique feature of the Bangladeshi COVID-19 vaccination timeline is abrupt peaks of greater than two vaccine doses per person administered during the first 6 months of 2022. In contrast, vaccine coverage in OECD member countries show a bi-modal profile with a peak of one dose vaccine reached in 2021 and a second peak of one to two doses of vaccine per person at the beginning of 2022. In Bangladesh, the reporting on vaccine doses was interrupted by gaps, whereas the OECD countries gave continuous daily updates on vaccination coverage [23]. On 1 March 2023, the Government of Bangladesh’s vaccine tracker data reported that 89 percent of the population had received 1 dose, 82 percent had received 2 doses and 48 percent had received a booster dose of the COVID-19 vaccines [2].

## 3. Human Behavior in the COVID-19 Pandemic

The use of rapid non-peer reviewed online preprint publications such as the cold spring harbor bioRxiv and the World Wide Web databases led to instant information on the immunology and molecular epidemiology of SARS-CoV-2 infection [30,31,32]. Early in the pandemic, spurious reports of anti-malarial and anti-parasitic drugs with inhibitory activity on coronavirus invasion and replication prompted clinical trials of chloroquine and hydroxychloroquine in COVID-19 patients. The numerous randomized trials showed no reduction in COVID-19-related mortality and no beneficial effects [33,34]. Widespread purchasing of chloroquine led to shortages of the medications and the inability of pharmacies to fulfill routine prescriptions for rheumatology patients [35]. In July 2020, a medical team from the Bangladesh Medical College Hospital sponsored by Beximco Pharmaceuticals’ managing director Nazmul Hasan in Dhaka claimed that a combination of the antiprotozoal medicine ivermectin and the antibiotic doxycycline was effective for the treatment of sixty COVID-19 patients, as “98% of patients had recovered within 4–14 days” [36]. Beximco Pharmaceuticals was later instrumental in launching the world’s first generic remdesivir, an antiviral drug for the treatment of COVID-19 patients developed by Gilead Sciences. These impulsive efforts of international medical science teams and the Bangladeshi public and private sectors to find a low-cost “quick-fix” to COVID-19 underscore the detrimental effect of hastily pursuing misguided goals based on anecdotal reports and populist beliefs rather than sound medical–scientific evidence.

In high-income countries, self-proclaimed experts and politicians disseminated misinformation on COVID-19 prevention and treatments via social media, which eroded public trust in peer reviewed publications on and clinical trials of COVID-19 treatments and vaccinations. The evidence-based recommendations of social distancing and wearing facial masks [37,38] were viewed with skepticism and, in some cases, despised by individuals aligned with populist agendas [39,40,41]. The guidelines of public health authorities and government organizations asking individuals to quarantine in the event of symptomatic COVID-19 or self-isolate after positive diagnosis via a RT-PCR of viral RNA or positive detection of N-protein antigens via rapid point-of-care tests were essential for controlling the spread of SARS-CoV-2 infection. Unfortunately, non-compliance with these preventive measures by large numbers of people and entire communities in North America, the United Kingdom and Europea coincided with irregular test reporting and inconsistent rules on days of quarantine and travel restrictions [40,41]. These factors contributed to increased viral transmission and the emergence of new SARS-CoV-2 variants in susceptible immune-suppressed patients or hospitalized cases of high-risk patients, with individuals experiencing chronic inflammation, malignancy, lung and or cardiovascular disease being susceptible to COVID-19.

Previous reviews give scathing criticisms of the Bangladeshi Government’s handling of the COVID-19 pandemic. Delays in testing, slow responses to quarantine, unethical stewardship and poor policies on the part of Bangladeshi health ministry were attributed to inherent government corruption including COVID-19 testing scams [42,43]. These negative impacts on the response to the COVID-19 pandemic were not unique to Bangladesh. The behavior of Bangladeshi government officials was no worse than that of the politically motivated misinformation and policy malpractice pervasive in the democratically elected representatives of high-income countries [40,41,42,43]. In this respect, negative human behavior worldwide was endemic prior to and during the emergence of SARS-CoV-2 variants and likely contributed to COVID-19-related morbidity and mortality among susceptible groups.

## 4. The SARS-CoV-2 Curriculum Vitae

To navigate the global public health threat of the COVID-19 pandemic, the WHO identified five major variants of concern (VOCs), alpha, beta, gamma, delta and omicron, defined according to increased rate of transmissibility, increased virulence or clinical disease symptoms or a negative impact on the efficacy of vaccines and therapeutics in preventing death or hospitalization due to severe illness [44,45,46,47]. The SARS-CoV-2 S-protein mutations have been described by Focosi et al. as “an accelerated movie of Darwinian natural selection” [48] whereby the number of key mutations acquired in the S-protein RBD correlates well with increasing fitness.

In March 2020, the SARS-CoV-2 strains showed limited adaptation to the human angiotensin-converting enzyme receptor hACE2, the receptor that SARS-CoV-2 uses for cell entry [49]. In 2021, base-pair mutations and recombinations were tracked using 15 million near-real-time genome sequence submissions in the EpiCoV database of the Global Initiative on Sharing All Influenza Data (GSAID) [50]. The first 18 months of the COVID-19 pandemic were characterized by the convergent evolution of mutations of SARS-CoV-2 S protein at residues K417, L452, E484, N501 and P681 across different variants of concern alpha, beta, gamma and delta [48,49]. Between February 2020 and May 2020, the variants carrying amino acid mutations from aspartate to glutamate at the spike protein position 614 (D614G variant) spread throughout the world and increased the transmissibility of SARS-CoV-2 by conferring higher viral loads in young hosts without an apparent increase in the severity of the disease [49,50,51]. In September 2020, new genetic variants, carrying E484K and N501Y mutations such as B.1.1.7 (also known as the UK variant) and B.1.351 (also known as the South African variant) showed greater transmissibility and capacity to escape antibody detection [52]. The delta variant (B.1.617.2), discovered in October 2020, was designated as a VOC by the WHO on May 11, 2021. The enhanced transmissibility of the delta variant has been associated with critical mutations such as D614G, L452R, P681R and T478K in the S protein [53,54]. In November 2021, the omicron and the sub-variants BA.1 and BA.2 in England rapidly replaced former SARS-CoV-2 strains to dominate the COVID-19 pandemic [55]. In Spring 2022 and throughout the third year of the pandemic, the omicron variant and its sub-lineages acquired additional groups of mutations at different amino acid residues, namely R346, K444, N450, N460, F486, F490, Q493 and S494 [51,56,57,58].

## 5. SARS-CoV-2 Variants Evolved Mechanisms of Host Cell Entry and Immune Evasion That Led to Superior Fitness over the Original Strain

The high frequency of mutations upon ACE2 binding and the polybasic furin cleavage site evolution of the spike protein led to enhanced rates of transmission of early variants relative to the hCoV-19/Wuhan/WIV04/2019 ancestral strain and likely indicated a viral genome of independent origins rather than transmission linked solely to a single source in the Huanan Seafood Market [44]. The cleavage of the S1–S2 boundary by the human transmembrane protease serine 2 (TMPRSS2) is a prerequisite for the S2 protein to initiate the membrane fusion process required for SARS-CoV-2’s entry into human cells [59]. The crystal structures of the RBD and ACE reveal 20 residues of ACE2 and 17 residues from the RBD-forming networks of hydrophilic side-chain interactions. The spike protein amino acid mutations that affect the interactions between the concave surface between monomeric ACE and trimeric RBD in the delta variants retain the S1–S2 furin cleavage site rather than switching to reliance on alternative host metalloproteases such as cathepsins [60]. Paradoxically, after furin cleavage, the S1 protein is prone to shedding and the SARS-CoV-2 is less infectious than previous coronaviruses. Several expert studies indicate that the D614G mutation compensated for the destabilizing effect of S1–S2 cleavage by increasing the strength of intermolecular associations between the S1 and S2 subunits. The increased infectivity of the D614G mutation is likely explained by the S1 structure having reduced shedding and RBD conformational change more favorable for host membrane fusion [59]. Omicron variants carrying the P681R mutation show comparable furin engagement and cleavage to the delta variant but utilize cathepsin proteases for cell fusion and viral entry [60]. The SARS-CoV-2 omicron sub-variants harbor highly mutated S proteins that confer the immune escape of nAbs without compromising the mechanisms of cell entry [61,62].

## 6. COVID-19 in Bangladesh—A Timeline of Infection, Morbidity, Deaths and Vaccination Coverage

The SARS-CoV-2 variants and the rates of vaccination, COVID-19 hospitalization and COVID-19 mortality in Bangladesh are compared at different time periods during the pandemic (Table 1).

The successive waves of SARS-CoV-2 infection in Bangladesh were driven by the alpha, beta, delta and omicron (BA.2, BA.4/.5, XBB) variants. Bangladesh reported its highest daily COVID-19 cases during these waves; 16,230 on 28 July 2021 and 16,033 on 25 January 2022 [2]. The third wave in the country, attributed to the delta variants, occurred between June 2021 and November 2021 [63,64], when Bangladesh reported its highest case fatality ratio (CFR), 2 percent, and highest rate of hospitalization, with a range of 47–59 percent ICU bed occupancy (Table 1). The Bangladeshi COVID-19 CFR of 2 percent and two-dose vaccination coverage of 10 percent during the peak period of the delta variant was like that of the COVID-19 CFR reported during the peak COVID-19 delta variant period in the North American and European populations pooled, which had CFRs of 2.3 (95% CI 0.17–4.84) and a two-dose vaccine coverage median of 31 percent [65]. The young age distribution of the Bangladeshi population, with 50% being less than 25 and 94% less than 65 years old, likely explains the country’s similar CFR to that of the overall CFR of high-income countries and underscores the need to compare COVID-19-related age-specific mortality between low-income and high-income countries. The emergence of a highly transmissible delta variant of severe COVID-19 sparked heightened anxiety and fear in Bangladesh [64], with a draconian response from the Bangladeshi Government [63]. The delta variant surge in India between February and June 2021 gave 400–600 daily deaths, 400,000 total deaths and excess mortality associated with the widespread scarcity of hospital beds, the lack of oxygen supply for critical patients and severe opportunistic fungal infection [66]. Bangladesh sealed its land border with India, implemented lockdowns and strict quarantine rules and ramped up its nationwide vaccination program [63]. These measures may have helped to delay the spread of the delta variant and reduce mortality in the COVID-19 third wave.

The slow start to the roll out of vaccinations to the Bangladeshi general population after the introduction of the first vaccine doses of Covishield to Dhaka health care workers in February 2021 did not translate into increased COVID-19-related mortality [25]. This report is consistent with the cemetery burial registrations in urban areas among Bangladesh’s predominantly Muslim population, an indicator of COVID-19-related mortality independent of hospital CFRs, which found that no significant excess deaths were observed during the delta variant surge compared to the period in 2020 at the onset of the COVID-19 pandemic [67].

The high rates of SARS-CoV-2 natural infection in the districts surrounding the Dhaka area prior to the introduction of the vaccination likely contributed to protective immunity in the young population. Our study on SARS-CoV-2 exposure between May and November 2020 revealed 25 to 29 percent N-protein IgG positivity in Dhaka and the highest SARS-CoV-2 seropositivity of 73 percent in Narayanganj, an urban district adjacent to Dhaka and located 30 km southeast, where the first COVID-19 cases were reported in March 2020 [68]. The high mobility of migrant workers and school children in Dhaka and Narayanganj, tracked using Facebook and mobile phone apps, established these locations as COVID-19 hotspots in Bangladesh. The digital portal also enabled the tracking of individuals, collection of nasopharyngeal swab specimens and genomic surveillance of SARS-CoV-2 in April 2020 [69].

In the period between May and June 2021 in Dhaka, 70 to 80 percent anti-N-protein IgG positivity and high levels of anti-N-protein IgA and anti-S-protein IgG were detected; this period was the onset of the delta variant surge [68]. The findings indicate prior exposure to SARS-CoV-2 with ongoing natural infection at a time when 10 percent of the Bangladeshi population had received two doses of vaccine.

Molecular surveillance of SARS-CoV-2 variants in Bangladesh between 26 May and 6 June 2021 revealed that 14 percent were alpha or beta variants, 11 percent were other variants and 75 percent were delta variants [70]. The symptoms of dry cough, difficulty breathing and oxygen support were found at higher frequencies among cases of the delta variants than among cases of the alpha and beta variants. The expansion of SARS-CoV-2 omicron variants into Bangladesh at the beginning of 2022 was associated with COVID-19 having ostensibly mild symptoms and no hospitalization, as well as 88 of 94 (94 percent) subjects receiving two vaccine doses [71,72].

## 7. SARS-CoV-2 Variants in Bangladesh—Life-Threatening Menace or Mild Problem?

**Figure 1:**
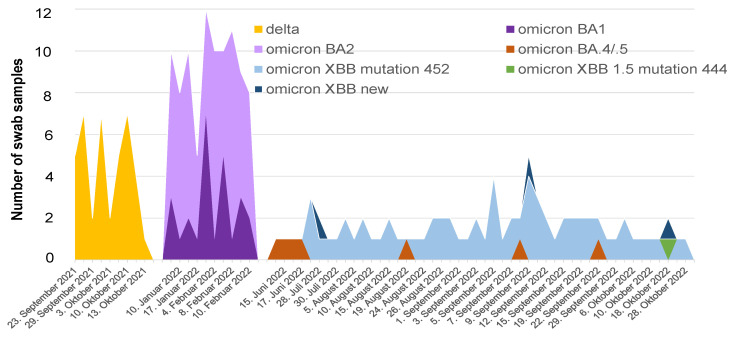
Timeline of circulating SARS-CoV-2 variants in Bangladesh. The delta variant (orange) n = 40 and omicron sub-variants BA.1 (dark purple) and BA.2 (light purple) n = 90 were identified from nasopharyngeal swab collections previously reported in Dhaka [70,71]. The omicron BA.4/BA.5 (brown) and omicron XBB (light blue) and novel sub-variants of XBB (dark blue) and BA2.75 (green) were identified from nasopharyngeal swab collections in Chittagong n = 31 and Dhaka n = 47, In Chittagong 19 percent had a previous SARS-CoV-2 infection, 84 percent had vaccine coverage (≥2 doses) and the interval between the date of collection and date of last vaccine dosage was 116 to 328 days. In. Dhaka: 32 percent had a previous SARS-CoV-2 infection, 94 percent had vaccine coverage (≥2 doses) and interval between the date of collection and date of last vaccine dosage was 151 to 500 days [76].

The first complete genome sequence of SARS-CoV-2 (CHRF_nCoV19_0001) from a Bangladeshi isolate was submitted to the GISAID database on 12 May 2020 from the Bangladesh-based Child Health Research Foundation [73]. The EpiCoV GISAID database contains 7701 entries of SARS-CoV-2 sequences from Bangladesh, of which 2000 entries belong to the omicron VOC B.1.1.529 plus BA as of 15 March 2023 [49,74,75]. The entries of the XBB N = 11 and XBB.1 omicron variants, N = 194, show the earliest collection dates on 12 September 2022, and 19 September 2022, respectively [50]. In sampling of SARS-CoV-2-positive nasopharyngeal swabs from Chittagong and Dhaka, we detect the omicron XBB variant on 28 July 2022, with the XBB and XBB sub-variants already predominant in August 2022 (Figure 1), and four unique Bangladeshi omicron sequences have been submitted to GISAID: EPI_ISL_17268140, EPI_ISL_17268138, EPI_ISL_17268139 and EPI_ISL_17268137 [76]. The XBB omicron is a recombinant of the BA.2.10.1 and BA.2.75 sub-lineages that was first documented on samples collected on 13 August 2022 [59]. In 2023, the rapid global spread of XBB variants was attributed to the F486P mutation in the S1 protein, which conferred increased binding affinity to the human cell receptor for SARS-CoV-2, ACE-2 [67], and multiple substitutions in the spike protein that cooperatively contribute to the resistance of XBB variants to humoral immunity [5,77]. Our findings for XBB in July and August 2022 in Bangladesh possibly indicate that XBB with mutations at spike protein amino acid 452 emerged in Bangladesh one to two months earlier than the documented dates of XBB and XBB.1 in India and Singapore [76].

The trend lines for the number of tests and the percentage of RT-PCR-confirmed COVID-19 were tracked to compare testing rates during the different periods of nasopharyngeal swab collections and SARS-CoV-2 variant reporting.

**Figure 2:**
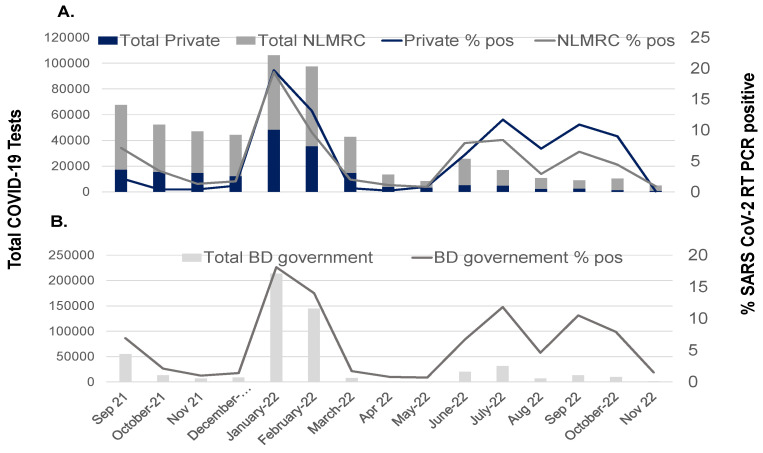
Timeline of suspected COVID-19 cases and percentage of cases testing SARS-CoV-2 RT-PCR-positive. Data from the Dhaka National Laboratory Medical Referral Center (NLMRC) or a Dhaka-based private laboratory (**A**) versus the Bangladeshi Government dashboard (**B**).

The records from one private laboratory in Dhaka and one public institute, NLMRC (Figure 2A), are consistent with the plotted data downloaded from the Bangladesh Government dashboard (Figure 2B). A stark decline in the percentage of SARS-CoV-2 RT-PCR positivity was observed at the end of delta variant wave in October 2021 and dropped below 2 percent positivity in November and December 2021. A rapid increase in SARS-CoV-2 testing, approximately 3-fold, was observed during the first omicron outbreak on 1 January 2022, which reached a peak RT-PCR positivity rate of 10–15 percent in February 2022 and then rapidly declined to the baseline testing rate of below 2 percent between 1 April 2022 and 31 May 2022. Two peaks in the COVID-19 testing volume were observed between 1 June 2022 and 31 July 2022 and between 1 August 2022 and 31 October 2022, corresponding to the subsequent waves of infection with the omicron sub-variants. Interestingly, both waves of omicron sub-variant infection are associated with a demonstrably low RT-PCR positivity rate of less than 3 percent. Infections with the SARS-CoV-2 omicron BA.1, BA.2, BA.4, BA.5 and XBB sub-variants report similar Cp values and viral loads among vaccinated and non-vaccinated cases and are associated with ostensibly mild or asymptomatic COVID-19 [55,76,77]. The most likely explanation for the low RT-PCR positivity rate during the period of SARS-CoV-2 omicron XBB detection in Bangladesh is that the Dhaka laboratories received an increased number of swab samples from non-COVID-19 respiratory infections compared to earlier periods due to new testing facilities and the expansion of testing capacity. Another reason is the government’s easing of international travel restrictions for Bangladeshis and the requirement for COVID-19 traveler reports. The screening of the general population, including asymptomatic individuals, revealed a low positivity rate.

The menace of severe COVID-19, highlighted by hospitalization and deaths during the delta variant surge, versus the mild or asymptomatic COVID-19 cases associated with successive waves of highly transmissible SARS-CoV-2 omicron sub-variants indicates that the virus evolved rapidly to evade immunity from natural infection and vaccination. The current circulation of antibody-evasive SARS-CoV-2 of low virulence and pathogenicity cannot predict the long-term trajectory of variants and whether the future cases of COVID-19 will be without severe illness or fatalities. The optimistic signs that COVID-19 is no longer a public emergency of international concern do not preclude the risk of new variants emerging to pose a health threat. Complacency and popular perception that the “COVID-19 pandemic is over” should not undermine the need for molecular surveillance of SARS-CoV-2 variants in animal reservoirs and the human populations of different countries.

## 8. Challenges and Lessons

Prior to and during the COVID-19 pandemic, the Bangladesh Institute on Tropical Infectious Diseases (BITID) in Chattogram and the NILMRC in Dhaka received training on protocols for sample handling and the evaluation of serological methods and non-routine molecular diagnostics of RNA viral infectious diseases. The levels of specific IgM and IgG and circulating viral antigens in the blood of patients with hepatitis E virus [78], dengue virus 68] or SARS-CoV-2 [70,71,76] infections were measured using newly developed ELISA kits, and the viral RNA was quantified via specialized real-time reverse transcriptase–polymerase chain reaction (RT-PCR) methods. The absence of bar-code systems and limited resources for the frozen storage of clinical specimens were major challenges that were partially overcome by prioritizing −80 °C freezer space, using special couriers for the pick up of dry ice from the Dhaka Linde facility and transport of cryovials from Germany. To minimize error in sample identification, the transfer of sera from blood collection tubes via cryovials and swabs into transport media involved teams working in pairs, with the same technician being responsible for the hand-written labeling of tubes. Improper labeling and poor sample quality did occur during periods of peak workload during the COVID-19 pandemic, but these exceptions were mostly resolved by core members of the team who were familiar with the protocols of handling viral RNA and sera established in projects prior to the pandemic.

Between May 2020 and November 2020, our studies revealed that the endemic dengue infections in Bangladesh prior to COVID-19 had no effect on the detection of antibody responses to SARS-CoV-2 N protein [68]. The materials from swabs in universal transport media and the serum samples were frozen at −80 °C and transported on dry ice for the quantification of viral RNA via post-RT-PCR analysis and the detection of S-protein mutations via mutation-specific (VirSNiPs) assays at TIB-MOLBIOL GmbH in Berlin, Germany. The abovementioned SARS-CoV-2 work plans were implemented during the early pandemic years of 2020 and 2021 when only a few laboratories in Dhaka were granted government approval for SARS-CoV-2 RT-PCR testing and imports of commercial immunoassays and rapid tests of SARS-CoV-2 were restricted.

The detection of SARS-CoV-2 in March 2020 was initially controlled and managed by the Bangladesh Institute of Epidemiology, Disease Control and Research (IEDCR) in Dhaka. In April 2020, a few members of the Virology Department of Bangabandhu Sheikh Mujib Medical University (BSMMU) and NILMRC worked relentlessly for 16 h per day to set up RT-PCR testing for SARS-CoV-2 in nasopharyngeal swab specimens of acute symptomatic COVID-19 cases using the collection tubes and reagents of the Sansure 2019-nCOV nucleic acid diagnostic kit and the thermocycler instruments, Applied Biosystems QuantStudio 5 and 7500 RealTime PCR System, Thermo Fisher Scientific, USA, provided by OMC Bangladesh Ltd., Dhaka There was sparse support for the pioneering team as colleagues were reluctant about having a testing facility due to the pervasive fear of spreading the contagious respiratory infection and the unknown outcomes of COVID-19 disease [79]. Gradually, the government and private firms started setting up their labs for testing. During the first 2 to 3 months, personal protective equipment (PPE) and PCR reagents were in short supply and very expensive. The WHO guidelines on isolation, tracing and treatment and the CDC infection prevention and control measures posed many challenges for implementation; in particular, the ventilation system in the Academic Department laboratories was not compliant with Biosafety level 3 (BSL-3). A four-storey office building space separate from the campus, and of old architectural design, was designated for COVID-19 testing. On the ground floor, booths were constructed for patients to enter after forming orderly outdoor queues, and on the first-floor, rooms were organized for specimen processing with rigorous precautions to avoid aerosol, as well as rooms for RNA isolation and reagent mixing and rooms with the instrumentation for amplification [80]. The facility received an average of 500 to 1000 samples per week, which increased five times over time, and reported test results were issued by text message to the patient’s mobile phone and sent daily to the management information system (MIS) under the Directorate General of Health Services (DGHS) that was added to the COVID-19 dashboard in 2020 [2]. As of 31 December 2020, there were 51 government laboratories and 63 private laboratories for COVID-19 testing in Bangladesh. By this date, the NILMRC and BSMMU reached 466,105 and 93,121 tests, respectively, which represented approximately sixty percent of the nationwide total; government laboratories performed 255,893 tests and private laboratories performed 669,667 tests. Between May 2020 and October 2020, the team’s know-how for setting up a COVID-19 testing facility was transferred to the NILMRC and the Dhaka private laboratory (Figure 2A).

The lesson learned in setting up PCR testing during the COVID-19 pandemic was the importance cooperation and commitment of personnel at all levels; health care workers, laboratory technicians and skilled scientists are essential for a successful emergency response. The drawbacks of establishing COVID-19 testing in an old building were the lack of negative pressure rooms with controlled HEPA-filtered air flow and absence of onsite autoclaving and effluent decontamination, which limited the operations of the facility to the temporary period between April and December 2020. The budget to cover instrumentation and initial salaries were provided by the government at the onset of establishing the facility but were not sustainable for the long-term costs of maintenance and retaining skilled laboratory staff. The estimated cost of SARS-CoV-2 PCR testing in Bangladesh during the COVID-19 pandemic was sourced from the government laboratories: 0.86 Euro per test from a booth and 2.60 Euro per test from home collection. The costs of SARS CoV-2 PCR testing in private laboratories were 30 Euro per test from a booth and 34 Euro per test from home collections.

The triage of sanitized booths and digital testing in Bangladesh in June 2020 was supported by funds from Concern Worldwide United Kingdom [80] and represented an early response to increases in the efficiency and capacity of RT-PCR testing after the general public frustration of waiting several days to receive their test results from public and private laboratories early in the pandemic. These Bangldeshi efforts pre-dated the July 2020 launch in United States of an expensive emergency room point-of-care Sofia^®^ SARS Antigen fluorescence immunoassay; the availability in Bavaria, Germany, in March 2021 of free rapid N-protein antigen lateral flow immune chromatographic tests; and the final issuance of an EU digital COVID certificate, the “EU green pass”, on 1 July, 2021, which covered vaccination, testing and recovery.

A previous report on Bangladeshi COVID-19 testing claimed that private laboratories charging patients a fee led to a decline in PCR testing in July and August 2020, which would have hampered the country’s health care system’s response to the COVID-19 pandemic [81]. The accuracy of this criticism is questionable. Bangladesh relies primarily on out-of-pocket payments to cover health care costs, with only 2.5% of the population possessing health insurance. Bangladeshi household out of pocket expenses (OOPE) accounts for 60% to 80% of the financing of health services and medicines. The overcrowding of private and public hospital facilities by poor patients unable to pay has led to the trend of wealthy patients seeking health care outside of the country. This structural fault was well recognized decades prior to the COVID-19 pandemic [82,83]. During the COVID-19 pandemic, the Bangladeshi Ministry of Health gave government hospitals but not private hospitals a budget for the managing the costs and expenses of testing and patient care in the absence of any pre-existing insurance system. In private laboratories, the costs and expenses of SARS-CoV-2 testing were covered by OOPE of the fees charged to patients. The urgency to reduce the burden of OOPE on household income involves ongoing discussion and financial plans for progressive reforms of the Bangladeshi health system.

In 2020, the warning of the worst scenario unfolding, “increased COVID-19 mortality during the monsoon and Dengue fever season”, raised concern and fear [84], which fortunately was proven wholly incorrect. The lesson learned during this infectious disease pandemic is that uncertainties regarding severe disease and case fatality ratios should be embraced by the focused and coordinated efforts of both private and government resources to ramp up diagnostic testing and vaccination. Surprisingly, this lesson holds true not only in high-income countries but also in densely populated low-income countries. The draconian lockdown measures of the Bangladesh Government’s initial response to the uncertainties of COVID-19, insufficient testing and inadequate personal protective equipment were quickly followed by impressive implementation of testing and dynamic mobilization of international funding for vaccinations. The management of the pandemic in Bangladesh was generally successful, considering the country’s strained health care resources and limited access to new technologies, modern equipment and skilled technical staff at laboratory facilities.

## 9. Conclusions

The legacy of COVID-19 pandemic is a multifaceted impact on human life and an unprecedented international response to a shared global predicament. Open access to information fascillitated understanding of SARS-CoV-2 infection and the COVID-19 illness. Government dashboards reported on the rates of RT-PCR positivity, hospitalization, COVID-19-related morbidity and mortality and vaccine coverage. Centralized data bases, such as the John Hopkins University Coronavirus Resource Center [1], the WHO COVID-19 Research Database, COVID-19 world maps [29,49,51], the Bangladesh Government dashboard [2,33] and the scientific resources of the Phylogenetic Assignment of Named Global Outbreak Lineage (PANGO) and GISAID [50,67], provided instantaneous and reliable updates on the COVID-19 pandemic. These objective online sources were no substitute for human compassion and motivation. The knowledge and expertise of skilled medical staff and scientists within communities and countries, as well as their openness to international collaboration, were the main drivers to find solutions for COVID-19 prevention and diagnosis and the treatment of SARS-CoV-2 infections.

COVID-19 caused major disruptions to socio-economic life, education and mobility irrespective of a country’s geography and political system. Medical researchers and health care professionals from countries with disparate resources, population densities, climates, cultures and religions were open to connecting for scientific collaboration, while they struggled to cope with the common challenges of uncertain SARS-CoV-2 infection and COVID-19 illness. The need to carry out systematic blood collections and nasopharyngeal swabs for serology and molecular surveillance was recognized as a priority by phyicians and laboratory staff worldwide.

Despite the initial challenges regarding inequities in vaccine distributions and cold-chain storage and transport, low-to-middle-income countries in Asia gained access to supply chains from the COVID-19 Vaccine Delivery Partnership (CoVDP) and benefited from funds related to COVID-19 relief and made important contributions of prospective clinical specimens to monitor SARS-CoV-2 infection and vaccine-induced immune responses. Manufacturers in high-income countries profited from worldwide demand for different diagnostic platforms and vaccines but also contributed critical real-world data on the performance of point-of-care and automated testing, anti-SARS-CoV-2 drugs, vaccines and molecular survellance of viral variants.

The emphasis on positive concerted efforts, rather than criticisms of inadequate government stewardship in a time of divisive political discourse, should be encouraged. The importance of an international mindset of helping cannot be taken for granted in future pandemic preparedness.

## Figures and Tables

**Table 1:** SARS-CoV-2 variants and COVID-19 in Bangladesh. COVID-19 deaths, ICU hospital bed occupancy and vaccinations are calculated for each period as a percentage of the total confirmed COVID-19 cases and vaccine doses reported in the Bangladesh Government dashboard database and the 2021 UN Census of Bangladesh’s 169 million population. The color-coded variants of concern (VOCs) are Wuhan; alpha; beta; delta; omicron BA.1, omicron BA.2, omicron BA.4 and omicron BA.5; and omicron XBB. O refers to other SARS-CoV-2 variants, and **x** refers to the n = 11 Bangladeshi XBB sequences carrying amino acid mutations in N proteins and S proteins listed in the GISAID EpoV database, 15 March 2023 [50].

COVID-19 Pandemic Period	SARS-CoV-2				Vaccination			COVID-19	
	Dominant VOCs				% Population Receiving Doses			Hospital Bed Occupancy	Deaths
					1	2	≥3	% ICU Beds	Nr (%)
April 2020–January 2021	Wuhan-like				0	0	0	NA	8050 (1.5)
February 2021–March 2021	Alpha			O	3.2	0	0	NA	991 (1.27)
April 2021–May 2021			Beta		3.4	2.46	0	47.6	3573 (1.9)
June 2021–September 2021			Delta		19.8	10	0	57.8	14,891 (2)
October 2021–December 2021					60.5	40.5	0.03	15.7	560 (1.9)
January 2022–February 2022			BA.2		90.2	59.8	2.3	20	965 (0.27)
March 2022–May 2022					93	79	9	6.1	93 (0.94)
June 2022–August 2022	BA.4/.5		XBB		93.7	81.8	25.8	5.9	193 (0.33)
September 2022–November 2022	**xxxxxxxxxxx**				100	84.2	35	4.6	110 (0.44)
December 2022–February 2023					100	91.2	41.6	3.1	12 (0.97)

## Data Availability

All of the sequencing data and information for this study are available in the GISAID database. The accession numbers are provided.
